# Achieving high coverage of larval-stage mosquito surveillance: challenges for a community-based mosquito control programme in urban Dar es Salaam, Tanzania

**DOI:** 10.1186/1475-2875-8-311

**Published:** 2009-12-30

**Authors:** Prosper P Chaki, Nicodem J Govella, Bryson Shoo, Abdullah Hemed, Marcel Tanner, Ulrike Fillinger, Gerry F Killeen

**Affiliations:** 1 Ifakara Health Institute, Coordination Office, Kiko Avenue, Mikocheni, PO Box 78373, Dar es Salaam, United Republic of Tanzania; 2 Liverpool School of Tropical Medicine, Vector Group, Pembroke Place, Liverpool, L3 5QA, UK; 3 Durham University, School of Biological and Biomedical Sciences, South Road, Durham, DH1 3LE, UK; 4 Dar es Salaam City Council, Ministry of Regional Administration and Local Government, United Republic of Tanzania; 5 Department of Public Health and Epidemiology, Swiss Tropical Institute, Basel, Switzerland; 6 London School of Hygiene and Tropical Medicine, Disease Control & Vector Biology Unit, Keppel Street, London, WC1E 7HT, UK

## Abstract

**Background:**

Preventing malaria by controlling mosquitoes in their larval stages requires regular sensitive monitoring of vector populations and intervention coverage. The study assessed the effectiveness of operational, community-based larval habitat surveillance systems within the Urban Malaria Control Programme (UMCP) in urban Dar es Salaam, Tanzania.

**Methods:**

Cross-sectional surveys were carried out to assess the ability of community-owned resource persons (CORPs) to detect mosquito breeding sites and larvae in areas with and without larviciding. Potential environmental and programmatic determinants of habitat detection coverage and detection sensitivity of mosquito larvae were recorded during guided walks with 64 different CORPs to assess the accuracy of data each had collected the previous day.

**Results:**

CORPs reported the presence of 66.2% of all aquatic habitats (1,963/2,965), but only detected *Anopheles *larvae in 12.6% (29/230) of habitats that contained them. Detection sensitivity was particularly low for late-stage *Anopheles *(2.7%, 3/111), the most direct programmatic indicator of malaria vector productivity. Whether a CORP found a wet habitat or not was associated with his/her unfamiliarity with the area (Odds Ratio (OR) [95% confidence interval (CI)] = 0.16 [0.130, 0.203], P < 0.001), the habitat type (P < 0.001) or a fence around the compound (OR [95%CI] = 0.50 [0.386, 0.646], P < 0.001). The majority of mosquito larvae (Anophelines 57.8% (133/230) and Culicines 55.9% (461/825) were not reported because their habitats were not found. The only factor affecting detection of Anopheline larvae in habitats that were reported by CORPs was larviciding, which reduced sensitivity (OR [95%CI] = 0.37 [0.142, 0.965], P = 0.042).

**Conclusions:**

Accessibility of habitats in urban settings presents a major challenge because the majority of compounds are fenced for security reasons. Furthermore, CORPs under-reported larvae especially where larvicides were applied. This UMCP system for larval surveillance in cities must be urgently revised to improve access to enclosed compounds and the sensitivity with which habitats are searched for larvae.

## Background

Historically, most vector control efforts for malaria prevention in Africa have focused almost exclusively on adult stages, specifically indoor residual spraying (IRS) [1,2] and insecticide-treated nets (ITN) [3-5]. However, with increasing insecticide resistance [6] and behavioural avoidance by mosquito vectors [7], development and evaluation of complementary vector control strategies remains a priority. Reviews of the early 20^th ^century programmes in Brazil, Zambia and Egypt [8-10], have highlighted dramatic reductions of malaria burden achieved by integrated vector management generally and mosquito larval control specifically [11-14]. Application of microbial larvicides, such as *Bacillus thuringensis *var. *israelensis *(*Bti*), to larval habitats offers a control option that cannot be avoided by mosquitoes [15,16] and that has low probability of developing resistance due to the complex mode of action of the larvicide [17,18]. Furthermore, recent successes in urban Tanzania [19], the highland of western Kenya [20] and in Eritrea [21], suggest that larval control may be a valid option for malaria vector control in selected eco-epidemiological settings.

Rapid growth of cities, characterized by a distinctive mix of different social, economic and cultural conditions is an important feature of contemporary African countries [22-25]. High population density associated with relatively few breeding sites suggests that area-wide application of vector control strategies is more practical and affordable in urban areas [26,27]. Moreover, stronger institutional support, governance and infrastructure offer significant advantages for establishing and sustaining vector control programmes in urban areas. However, the heterogeneity and mobility of the human population renders most urban communities less cohesive and therefore difficult to mobilize *en masse *to achieve impact of public health interventions. Malaria vector proliferation, transmission intensity and burden in urban areas is highly heterogeneous and focal, [23,26,28-30]. Despite its growing importance, it is only recently that urban malaria is receiving the attention it deserves [23,25,26].

Cities and large towns are regarded as some of the most favourable environments for sustainable mosquito larval control, because mosquito-breeding sites are defined and easily located. However, larval control requires quite specific ecological understanding of the major vector species and their distinctive interaction with the local environment on very fine spatial scales [11,31,32]. Additionally, technical understanding of the principles and practice of larvicide application or environmental management, as well as intensive labour under challenging field conditions, are essential [11,31-33]. Sustainable systems for monitoring the abundance and distribution of aquatic mosquito stages are required to enable effective decisions and actions by managers responsible for such programmes. This represents a particular challenge in Africa where the primary vector, *Anopheles gambiae*, can develop from egg to adult in less than a week in habitats, which can be ephemeral and difficult to detect [34-36].

Larval control for malaria prevention, delivered primarily through human resources mobilized from within local communities, has been recommended to minimize cost and maximize sustainable scalability [31-33,37]. However, given the technical, logistic and coverage requirements of larval control, which are probably greater than for current priority measures, such as insecticide-treated nets or indoor residual spraying, community-led rather than merely community-based vector control may be difficult to achieve [31,35,37]. A more sustainable approach might be the blending of vertical and horizontal strategies for the implementation of community-based systems for delivering area-wide control measures. Such an approach might rely on extensive mobilization of community-based labour integrated into vertical management systems implemented by centralized institutions [31,35,37]. It is important to identify and understand the social and environmental factors that influence human behaviour and consequently the effectiveness of such programs.

The Urban Malaria Control Programme (UMCP) in Dar es Salaam has been initiated by the Dar es Salaam City Council as a pilot programme to develop sustainable and affordable systems for larval control as part of routine municipal services [19,32,35,37-39]. An in-depth look at the environmental and programmatic determinants of surveillance coverage in this urban environment was conducted to identify strengths, weaknesses and opportunities for improvement.

## Methods

### Study area

Dar es Salaam is Tanzania's biggest and most economically important city with the current population size exceeding 2.5 million inhabitants and a total area of 1,400 km^2^, corresponding to a mean human population density of 2,900 per km^2 ^[40]. It is situated between latitude 6.0°-7.5° S and longitude 39.0°-39.6° E. The city is divided into three municipalities: Kinondoni, Temeke and Ilala and each of these municipalities is further divided into wards. The study site comprised the 15 wards with 614,000 residents [40] included in the Dar es Salaam UMCP, [7,19,32,37] covering an area of 55 km^2 ^with wards ranging in size from 0.96 to 15 km^2^. All UMCP activities are coordinated by the City Medical Office of Health, and are fully integrated into the decentralized administrative system in Dar es Salaam, operating on all six administrative levels of the city: the city council, the three municipal councils it oversees, 15 wards chosen from those municipalities, containing 67 neighbourhoods referred to as *mitaa *in Kiswahili (singular *mtaa*, meaning literally street), and more than 3,000 housing clusters known as ten-cell-units (TCU) with each of them subdivided into a set of plots corresponding largely to housing compounds. The main tasks on the three upper levels are programme management and supervision, whereas mosquito larval surveillance and control is organized at ward level and implemented at the level of TCUs and their constituent plots. In principle, a TCU clusters ten houses with an elected representative known as an *mjumbe*, but typically comprises between 20-100 houses in practice [41]. UMCP implements regular surveillance of mosquito breeding habitats as a means to monitor effective coverage of aquatic habitats with microbial larvicides. Surveillance is applied through a community-based [35] but vertically managed delivery system [37]. The cross-sectional surveys described here to evaluate routine surveillance activities were conducted between end of June 2007 and January 2008. This period spanned a full dry season and was preceded by a typical rainfall pattern with a main rainy season from March to June and a much shorter rainy season from October to December.

### Routine programmatic larval surveillance by community based personnel

Community owned resource persons (CORPs) were recruited through local administrative leaders including Street Health Committees and were remunerated at a rate of 3,000 Tanzanian shillings (US$ 2.45) per day through a casual labour system formulated by the municipal councils of Dar es Salaam for a variety of small-scale maintenance tasks such as road cleaning and garbage collection [32,35]. All essential standard operating procedures adopted by the recruited larval surveillance CORPs are described in detail elsewhere [37], but summarized as follows.

Over 90 larval surveillance CORPs were actively employed by the UMCP during the time of survey with each assigned to a defined area of responsibility, comprising a specific subset of TCUs within one neighbourhood. These lists of TCUs were initially allocated based on local knowledge of habitat abundance, difficulty of terrain and geographic scale and subsequently refined through detailed participatory mapping of the study area [41]. On average, one CORP was responsible for an area of approximately 0.6 km^2^. All CORPs worked under the oversight of a single ward-level supervisor. Each CORP followed a predefined schedule of TCUs, which they were expected to survey on each day of the week. In wards where larviciding was taking place, the schedule of TCUs visited by the surveillance CORPs followed one day after the application of microbial larvicides by a separate set of larval control CORPs [37] so that indicators of operational shortcoming, such as the presence of late-stage (3^rd ^or 4^th ^instar) mosquito larvae, could be reacted to in sufficient time to prevent emergence of adult mosquitoes. This system was designed for routine mosquito habitat surveillance and larviciding to allow timely interpretation and reaction to entomologic monitoring data.

### Qualitative preliminary assessment of community-based larval surveillance

The investigator (PPC) initially conducted three weeks of unscheduled guided walks with 23 of the surveillance CORPs nominated by the ward supervisor after the investigator reported to their office in the morning. The investigator did not pre-inform the CORPs nor did he reveal his role and independent status at any time before or during the visit. Both the investigator and the chosen CORPs would leave the ward office and survey TCUs that the CORPs were expected to survey according to their normal predefined schedule for that particular day [37], returning later to report to the ward supervisor. At this stage, the survey was led by the CORPs and the investigator followed passively, covertly observing and recording how CORPs conducted their routine larval habitat surveillance and prepared their daily reports for submission to the ward supervisor. Specifically, the following information was collected: did CORPs follow TCUs schedule correctly, were all TCUs and plots visited, whether fenced compounds were entered and if not, why not, how habitats were recorded, how habitats were searched for larvae, how CORPs interacted with residents. In cases of observed shortcomings in the operational practices of the CORPs or any additional opportunities for improved implementation of their duties, the CORPs were informally advised by the investigator. This approach was intended to maintain an open, non-authoritative relationship of the investigator with the CORPs, allowing him to observe and understand the operational challenges facing the CORPs and the program as a whole. A detailed formal analysis of these qualitative observations will be published elsewhere but informal appraisal of these observations was used to design a quantitative survey described as follows.

### Quantitative cross-sectional evaluation of community-based larval surveillance

A total of 173 TCUs from neighbourhoods distributed across all 15 wards were randomly selected from the list of TCUs in the UMCP study area. A total of 64 CORPs were responsible for these selected TCUs. The investigator accompanied the relevant CORP in guided walks through each TCU one day after their scheduled routine surveillance of that TCU and implemented his own larval habitat surveys following the standard operating procedures [37]. Results of the investigator were compared with the CORP's datasheet of the previous day. Every potential habitat found by the CORP in each plot, and any additional habitats identified by the investigator that had not been detected by the surveillance CORPs, were distinguished and recorded using standardized forms (Additional file 1). Habitats were further classified into three habitat categories and constituent 11 habitat types [35] as follows: (1) natural habitats comprising (i) marshy or swampy areas, (ii) river-beds and (iii) springs or seepages; (2) agricultural artificial habitats comprising (i) rice paddies, (ii) ridge and furrow agriculture (*matuta*) and (iii) other habitats associated with agriculture; (3) non-agricultural artificial habitats comprising (i) drains and ditches, (ii) construction pits, foundations and other excavations (iii) water storage containers, (iv) tyre tracks and puddles and (v) ponds or pools. Additional information was collected regarding the presence or absence of a fence around a plot and whether or not a particular TCU was targeted with larvicide application at the time that it was surveyed. Lastly, records were taken regarding evidence of lack of familiarity of a CORP with the specific TCU and plots. Unfamiliarity was assumed if the CORP was not readily able to find his or her way around the TCU or plot, when plot boundaries could not be clearly defined and/or when residents of the plot were unable to recognise him/her as a regular visitor to the area.

### Statistical analyses

All the data were entered in coded numeric form and analysed using SPSS 15.0. Any association between the occupancy of different mosquito habitat categories and types by *Anopheles *and Culicine larvae was analysed using multivariate binary logistic regression [42]. Specifically, generalized estimating equations (GEE) were fitted to determine the influence of lack of familiarity of the CORP with the area, presence of a fence around the plot and whether larviciding was operational in that time and place upon the proportion of wet habitats (detection coverage) reported by CORPs and the proportion of habitats which contained larvae that were reported to be occupied by the CORP (detection sensitivity) for different habitat categories or types. While all observed habitats were included in the model fits to assess detection coverage, only those found to contain larvae by the investigator were considered in the denominator of models to assess detection sensitivity. The detection of the wet habitat or larval occupancy by the CORP was treated as the binary outcome variable and was fitted to a binomial distribution with a logit link function. CORP identity was treated as the subject variable and an exchangeable correlation matrix chosen for the repeated measurements distinguished by plot identity as the within subject variable. Differences between frequency distributions were assessed using likelihood ratio χ^2 ^analysis.

## Results

### Habitat characteristics found during cross-sectional evaluation

**Figure 1:**
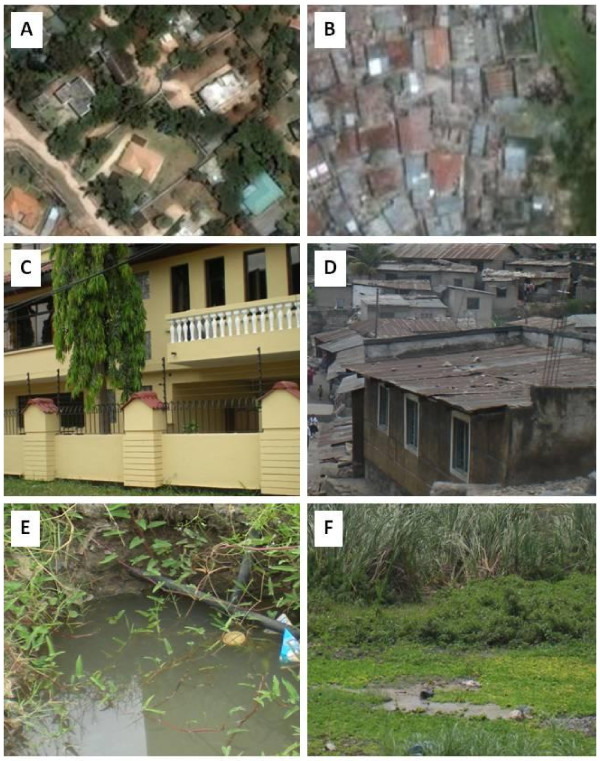
**Aerial photos for planned (A) and unplanned (B) settlements of urban Dar es Salaam with ground-based photos of common features for each (C and E versus D and F, respectively)**. Planned settlements are characterized by relatively wealthy inhabitants, fences, tight security and restricted access but often contain suitable habitat within spacious plots (**E **was photgraphed within the compound seen in from the ground in **C **and from the air in **A**). Unplanned areas are characterized by dense settlement, scarce space for habitats, almost no fences and few but often prominent habitats which are readily accessible (**F **is located at the bottom of the valley pictured from the ground in **D **and from the air in **B)**.

A total of 8,395 plots were visited during the cross-sectional surveys, 60.0% (5,039) of which were from larviciding areas (Figure 1). Approximately one quarter of these plots (26.8%; 2,253) was behind fences. There was an unequal distribution of fenced plots between the visited larviciding and non-larviciding areas with the majority of the fenced plots (69.7%; 1,571) recorded in areas where larviciding was taking place. Overall 3,997 potential mosquito breeding habitats were recorded. Ofthese, 2,965 (74.2%) contained water at the time of survey. The vast majority of these wet habitats were non-agricultural artificial habitats (90.0%), such as drains, ditches, construction sites, foundations, man-made holes and tire tracks. The remainder was composed of a small number of natural habitats (7.4%), such as swampy areas with high groundwater level, riverbeds, seepages and springs, and a few agricultural artificial habitats (2.6%) mainly associated with rice and sweet potato cultivation; crops grown in ridge and furrow systems known as *matuta *(Table 1).

**Table 1:** Occupancy of different mosquito habitat categories and types by all stages of *Anopheles *and *Culicine *larvae.

**Variables**	***Anopheles *larvae occupancy**			**Culicine larvae occupancy**		
		
	**Proportion occupied %** **(n/N)^a^**	**OR [95%CI]**	**P**	**Proportion occupied % (n/N)^a^**	**OR [95%CI]**	**P**
						
***Natural Habitats***	***28.64 (63/220)***	***1.00*^b^**	***NA*^b^**	***20.00 (44/220)***	***1.00*^b^**	***NA*^b^**
Marsh/swampy areas	36.25 (58/160)	1.00^c^	NA^c^	11.88 (19/160)	1.00^c^	NA^c^
Riverbeds	8.33 (2/24)	0.38 [0.09,1.64]	0.192	95.83 (23/24)	137.54 [18.17, 1041.38]	< 0.001
Seepages/springs	8.33 (3/36)	0.38 [0.11,1.26]	0.113	5.56 (2/36)	0.35 [0.08,1.51]	0.160
						
***Agricultural artificial habitats***	***43.42 (33/76)***	***1.91 [1.12,3.28]***	***0.019***	***22.37 (17/76)***	***1.15 [0.61, 2.17]***	***0.660***
						
Rice paddies	71.48 (5/7)	10.33 [1.96,54.39]	0.006	14.28 (1/7)	0.99 [0.12, 8.46]	0.998
Matuta	47.06 (16/34)	3.67 [1.78,7.57]	< 0.001	29.41 (10/34)	2.49 [1.12, 5.52]	0.025
Other agriculture	34.39 (12/35)	2.16 [1.02,4.55]	0.044	17.14 (6/35)	1.24 [0.49, 3.13]	0.653
						
***Non-agricultural artificial habitats***	***5.06(135/2669)***	***0.13 [0.09, 0.19]***	***< 0.001***	***28.81(769/2669)***	***1.60 [1.14,2.26]***	***0.007***
						
Tyre tracks/puddles	19.48 (68/349)	2.35 [1.55,3.57]	< 0.001	14.33 (50/349)	0.81 [0.46,1.42]	0.454
Drain	1.96 (21/1070)	0.84 [0.05,0.14]	< 0.001	20.84(223/1070)	1.60 [1.15, 2.24]	0.006
Construction sites	6.25 (42/672)	0.27 [0.18,0.41]	< 0.001	31.55 (212/672)	2.70 [1.92,3.80]	< 0.001
Water storage containers	0.34 (2/587)	0.01 [0.003,0.058]	< 0.001	47.36 (278/587)	5.34 [3.80, 7.51]	< 0.001
Ponds	18.18 (2/11)	0.92 [0.19,4.35]	0.914	54.55 (6/11)	7.18 [2.11, 24.40]	0.002
						
***Total***	***7.79(231/2965)***	***NA***	***NA***	***27.99(830/2965)***	***NA***	***NA***

The proportion of wet habitats found by investigator to contain *Anopheles *and Culicine larvae; Odds ratio (OR) and P values for the likelihood of occupancy determined with a binary logistic regression treating habitat category or type as potential determinants.

^a ^N is the total number of all wet habitats found during cross-sectional surveys while n is the number of either Anopheles or Culicine larvae positive habitats found

^b ^is the reference group for comparing habitat categories,

^c ^is the reference group for comparing the habitat types,

CI = confidence interval

NA; Not applicable

Almost half (45.6%; 1,351/2,965) of all aquatic habitats were located within fenced plots. One fifth (20.5%; 608/2,965) of all aquatic habitats were recorded in plots with which CORPs clearly appeared to be unfamiliar and 91.9% (539/608) of these were located behind fences. A large number of wet habitats were surveyed in both larviciding areas (1,895) and in non-larviciding areas (1,070) and the proportion of habitats within fenced plots was higher in areas with larviciding than those without (50.8% (962) versus 36.4% (389), respectively; χ^2 ^= 57.3, df = 1, P < 0.001).

Only 7.8% of all the surveyed habitats contained any aquatic stages of *Anopheles *larvae (Table 1) so there were relatively few habitats in which the sensitivity with which CORPs detected these key indicators of malaria vector proliferation could be assessed. Unexpectedly, three quarters (74.8%, 172/230) of anopheline-occupied habitats were found in larviciding areas and anopheline larval occupancy was twice as high in wards where larviciding took place as those without (9.1% (172/1,895) versus 5.5% (59/1,070); Odds Ratio [95% Confidence Interval] = 2.11 [1.20-3.67], P = 0.009). Overall, 7.0% (207/2,965) of wet aquatic habitats contained early-stage (1^st ^and 2^nd ^instars) *Anopheles *larvae, whereas 5.2% (155/2,965) of aquatic habitats were inhabited by late-stage *Anopheles *larvae (3^rd ^and 4^th ^instars), with 71.6% (111/155) of the latter recorded in areas with larviciding.

The probability of a habitat containing Anopheline larvae depended on category and habitat type (Table 1). Agricultural sites were twice as likely to contain Anopheline larvae than natural habitats, whilst the chance of finding larvae in artificial non-agricultural habitats was much lower. Nevertheless, non-agricultural artificial habitats were the most abundant (90%) and, therefore, constituted 58% (135/231) of all *Anopheles*-occupied habitats (Table 1).

Over one quarter of wet habitats contained culicine larvae (Table 1), with 25.9% (767) and 22.1% (656) inhabited by early-stage and late-stages respectively. Natural and agricultural habitats were equally likely to harbour culicine larvae whilst the probability of their presence was significantly higher in artificial, non-agricultural habitats (Table 1).

### CORPs' detection of aquatic habitats

CORPs recorded 1963 wet habitats during their routine surveillance. Seven of these habitats were confirmed to be non-existent by the investigator, suggesting these CORPs had filled the surveillance forms without visiting the relevant plots so these were excluded from the analyses. Therefore, CORPs correctly recorded two thirds of wet habitats (Table 2). Detection coverage varied significantly between individual CORPs and between different habitat types (P < 0.001 for both as determined by logistic regression). CORPs were unfamiliar with 20.5% (608) of wet habitats and 92% (539) of these were located behind fences. Furthermore, the majority of wet habitats that the CORPs failed to record (61.1%; 619/1009) were located within fenced plots.

**Table 2:** Detection efficiency of different

**Habitat Category**	**Habitat type**	**Total number of wet habitats detected by**		**Proportion detected by CORPs (%)**
			
		**CORPs**	**Investigator**	
				
Natural Habitats	Marsh/swampy areas	93	160	58.1
	Riverbeds	24	24	100.0
	Seepages	29	36	80.6
	***Subtotal***	***146***	***220***	***66.4***
				
Agricultural artificial habitats	Rice paddies	3	7	42.9
	*Matuta*	23	34	67.6
	Other agriculture	18	35	51.4
	***Subtotal***	***44***	***76***	***59.9***
				
Non-Agricultural artificial habitats	Tyre tracks/puddles	176	349	50.4
	Drains	898	1050	85.5
	Construction sites	450	672	67.0
	Water storage containers	231	587	39.4
	Ponds	11	11	100.0
	***Subtotal***	***1766***	***2669***	***66.2***
				
**Total**		**1956**	**2965**	**66.0**

CORPs; community-owned resource persons

**Figure 2:**
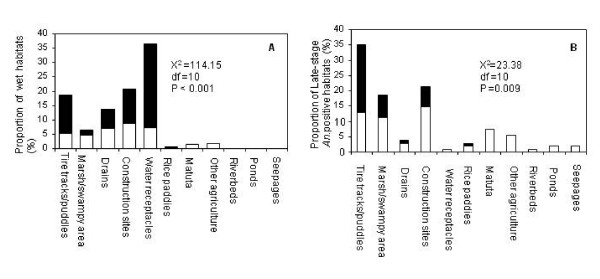
**Proportions of wet habitats (A) and late-stage *Anopheles*-positive habitats (B) found by CORPs within fenced (Black bars) and unfenced (White bars) plots**.

Detection coverage differed significantly for different habitat types (χ^2 ^= 432.8, df = 10, p < 0.001) and categories (Table 3) with artificial non-agricultural habitats 1.6 times more likely to be recorded than others (Table 3). Consistent with the baseline evaluation [35] conducted before the introduction of current procedures for mapping [41], surveillance and larvicide application [37], most conspicuous habitat types like ponds, rivers, seepages, springs and drains were more readily recorded, whereas water receptacles were poorly detected (Table 2). Furthermore, the type of habitats that CORPs did not find was significantly different between the fenced and unfenced plots: the majority of water storage containers, tyre tracks and artificial pits were located behind fences (Figure 2).

**Table 3:** Factors associated with habitat detection coverage by CORPs.

**Variable**	**% (n/N)**	**OR [95%CI]**	**P**
			
*Habitat category*	NA	NA	0.053
Natural	66.4 (146/220)	1.00^a^	NA
Artificial non-agricultural	66.1(1766/266)	0.60 [0.406,0.909]	0.015
Artificial agricultural	57.9 (44/76)	1.38 [0.607,3.143]	0.441
*CORPs familiarity with plot*	NA	NA	< 0.001
No evidence of unfamiliarity	75.8(1788/235)	1.00^a^	NA
Clear evidence of unfamiliarity	27.6 (168/608)	0.16 [0.130,0.203]	< 0.001
*Intervention status*	NA	NA	0.978
Non-larviciding	72.4 (775/1070)	1.00^a^	NA
Larviciding	62.3(1181/189)	0.99 [0.645,1.548]	0.997

The probability that a wet habitat was detected by the CORPs was modelled with a binary distribution and logit link function using Generalised Estimating Equations (GEE) treating intervention status, CORPs' unfamiliarity with the plots and habitat category as the potential predictors

^a ^the reference group for the particular variable,

CI; confidence interval,

CORPs; community-owned resource persons

N; the number of wet habitats found during cross-sectional surveys

n; the number of wet habitats found by the CORPs during their routine habitat survey,

NA; Not applicable

OR; Odds ratios,

The probability of a CORP detecting and recording a wet habitat was similar in larviciding and non-larviciding areas but was 84% less likely if he or she was unfamiliar with the area (Table 3). As mentioned earlier, the vast majority of the sites with which the CORPs were unfamiliar were within fenced plots. The covariance between these two variables (Pearson correlation, r^2 ^= 0.40, P < 0.001) implies that the presence of fences around plots contributed to the lack of familiarity with plots among CORPs. Although excluded from the selected model presented in Table 3 because of this covariance, fenced plots, selected when familiarity was excluded, reduced the detection coverage by half (OR [95%CI] = 0.49 [0.37-0.65], P < 0.001).

### CORPs' detection of aquatic stage mosquitoes

Overall detection sensitivity of mosquito larvae was very low among CORPs. They found only 29 of 230 anopheline-positive habitats and 263 out of 830 culicine-positive habitats, corresponding to under-reporting rates of 87.4% and 68.4%, respectively. CORPs reported a higher proportion of larva-containing habitats in non-larviciding areas (anophelines: 27.6% (16/58) and culicines: 44.4% (138/311)) than larviciding areas (anophelines: 7.6% (13/172) and culicines: 24.1% (125/519)). Detection sensitivity was twice as high for early instars 13.5% (28/207) than late instars 6.5% (10/155) of anopheline larvae (χ^2 ^= 4.72, df = 1, P = 0.029). Detection sensitivity for early and late-stage culicine larvae did not differ (32.2%, (247/767) and 30.0%, (196/653) respectively (χ^2 ^= 0.787, df = 1, P = 0.375). Not only did most habitats (71.6%; 111/155) that contained late-stage anophelines during the investigator's survey occur in the larviciding areas, CORPs had reported this indicator of mosquito proliferation in only 3 of these cases (2.7%). Detection sensitivity of late stage Anopheles in non-larviciding areas was also very low (15.9%; 7/44) and did not differ significantly (P = 0.124) from larviciding areas.

Failures to detect mosquito larvae can be attributed to two distinct causes: (1) the aquatic habitat was not found and therefore no larval search took place or (2) the larvae were not detected during the inspection of that habitat. More than half of the anopheline (57.8%; 133/230) and culicine-positive (56.0%, 465/830) habitats were not recorded as wet by CORPs. In 60.9% and 95.7% of these non-reported anopheline and culicine-occupied habitats, respectively, the CORPs was either unfamiliar (anophelines; 45.1%, (60/133), culicines; 52.5%, (244/465)), the plot was fenced (anophelines; 45.9%, (61/133), culicines; 64.3%, (299/465)) or both (anophelines; 30.1%, (40/133), culicines; 21.1% (98/465)).

Anopheline larvae were identified by CORPs in only 29 of the 97 occupied habitats which they recorded as wet so overall detection sensitivity was 29.9%. More importantly they appeared unfamiliar with very few of both the anopheline-positive habitats which they reported as wet (5.2%, 5/97) and those which they did not (7.4%, 5/68). It therefore appears likely that not reporting larvae is due to insufficient dipping, examination or training in mosquito identification rather than not visiting the site. Notably, the detection sensitivity for culicine larvae in habitats that were reported as wet was much higher with almost three quarters of habitats containing these more obvious larvae being successfully identified (Table 4).

**Table 4:** Detection sensitivity of larval stages in different aquatic mosquito larval habitat types and categories by CORPs

		**Anophelines**			**Culicines**		
		**Number of habitats found with larvae by^a^**		**Proportion detected by CORPs (%)**	**Number of habitats found with larvae by^a^**		**Proportion detected by CORPs (%)**
		**CORPs**	**Investigator**		**CORPs**	**Investigator**	
	
Natural Habitats	Marsh/swampy areas	5	24	20.8	10	13	76.9
	Riverbeds	1	2	50.0	18	23	78.3
	Seepages	0	1	0.0	1	2	50.0
	***Subtotal***	*6*	*27*	*22.2*	*29*	*38*	*76.3*
Agricultural artificial habitats							
	Rice paddies	0	2	0.0	0	1	0.0
	*Matuta*	4	9	44.4	7	9	77.8
	Other agriculture	1	5	20.0	0	1	0.0
	***Subtotal***	*5*	*16*	*31.3*	*7*	*11*	*63.6*
Non-agricultural artificial habitats							
	Tyre tracks/puddles	1	14	7.1	15	21	71.4
	Drains	7	14	50.0	122	165	73.9
	Construction sites	9	24	37.5	68	91	74.7
	water storage containers	0	0	0.0	14	32	43.8
	Ponds	1	2	50.0	6	6	100.0
	***Subtotal***	*18*	*54*	*33.3*	*225*	*315*	*71.4*
	Total	**29**	**97**	**29.9**	**261**	**364**	**71.7**

^a^out of those habitats that were recorded as wet by the CORPs during their routine surveys

Larval detection sensitivity was different for different habitat types for anophelines (χ^2 ^= 28.9, df = 10, P = 0.001) and culicines (χ^2 ^= 21.6, df = 10, P = 0.016). CORPs more readily detected anopheline larvae in larger, more obvious habitats like drains, riverbeds, ponds and *matuta *(Table 4). To enable fitting of a logistic model, these types had to be pooled into categories which had no significant effect. However, the probability of CORPs reporting larval anophelines occupying a habitat was drastically reduced if the habitat was located in an area where larviciding was ongoing (Table 5).

**Table 5:** Factors associated with Anopheline and Culicine detection sensitivity in wet habitats reported by CORPs.

		**Anophelines**			**Culicines**	
						
**Variable**	**% (n/N)**	**OR [95%CI]**	**P**	**% (n/N)**	**OR [95%CI]**	**P**
						
***Habitat category***	**NA**	**NA**	**0.331**	**NA**	**NA**	**0.421**
						
Natural	22.2 (6/27)	1.00 [NA]^a^	NA^a^	76.3 (29/38)	1.00 [NA]^a^	NA^a^
Artificial agricultural	31.3 (5/16)	2.03 [0.397-10.375]	0.395	63.6 (7/11)	0.72 [0.220-2.366]	0.590
Artificial non-agricultural	33.3(18/54)	2.34 [0.7607.231]	0.138	71.4(225/315)	1.39 [0.714-2.688]	0.336
						
***Intervention status***	**NA**	**NA**	**0.042**	**NA**	**NA**	**0.005**
Non-larviciding	40.0 (16/40)	1.00 [NA]^a^	NA^a^	80.6 (137/170)	1.00 [NA]^a^	NA^a^
larviciding	22.8 (13/57)	0.37 [0.142-0.965]	0.042	63.9 (124/194)	0.35 [0.167-0.722]	0.005

The probability of mosquito larvae detected by the CORPs modelled with a binary distribution and logit link function using Generalised Estimating Equations (GEE) treating intervention status and habitat category as the potential predictors.

^a^; reference group for particular variable

CI; confidence interval

CORPs; community-owned resource persons

N; the number of habitats that were reported to be wet by CORPs during routine habitat surveys and contained larvae during cross-sectional surveys

n; the number of habitats where CORPs found larvae during their routine habitat survey,

NA; Not applicable

Late-stage *Anopheles *occupancy was reduced by over 70% in habitats in the intervention areas where the surveillance CORPs actually found and reported the wet habitats (Table 6). Note that no significant reduction of late-stage *Anopheles *occupancy was revealed for habitats in areas not covered by the intervention, regardless of whether the surveillance CORPs found them or not (Table 6).

**Table 6:** Impact of larviciding on late stage *Anopheles *larvae occupancy.

**Variable**	**Proportion occupied % (n/N)**	**OR [95% CI]**	**P**
			
*Intervention status*			
Non-larviciding	4.1 (44/1070)	1.00^a^	NA
Larviciding area	**5.9(111/1895)**	**2.32 [2.19,6.14]**	**< 0.004**
***Intervention status × habitat found by CORP***			
*Found and reported by CORPs*			
Non-larviciding	0.9 (7/782)	1.00^a^	NA
Larviciding area	**0.3 (3/1181)**	**0.22 [0.147,0.34]**	**< 0.001**
*Not found or reported as dry habitats*			
Non-larviciding area	4.7 (37/782)	1.00^a^	NA
Larviciding area	9.1(108/1181)	0.73 [0.383,1.37]	0.325

The Odds of change of late Anopheles habitat occupancy subject to CORPs detection sensitivity of wet habitats and subsequent larvicide application as interacting terms modelled with a binary distribution and logit link function using Generalized Estimating Equations (GEE)

^a ^reference group for a particular variable,

CI; confidence interval,

NA; Not applicable

n/N; the proportion of all habitats found to contain late stage *Anopheles *larvae by observations of the CORPs and the investigator.

OR; Odds ratios.

## Discussion

The observation that CORP surveys at this stage of the UMCP's development had detected 66% of all aquatic habitats represents an improvement upon the 41% reported at the baseline surveys [35] but nevertheless leaves significant room for improvement. The majority of the habitats that were not reported by CORPs, including most of those containing larvae, could be attributed to CORPs' unfamiliarity and, most importantly, to the presence of a fence. The latter is one of the most prominent features in urban settings, presumably resulting from growing security challenges. Limited access to the fenced plots reduces the chances of habitats being found, reported or treated, and undermines coverage of surveillance and vector control activities. The fact that 75% of habitats with *Anopheles *mosquitoes that the CORPs did not find came primarily from three habitat types (puddles, marshes and construction sites), most of which were behind fences (30.3%), suggests considerable opportunity to achieve improvement through targeted training and increased emphasis upon these habitat types and plot characteristics (Figure 2). Notably, the CORPs more readily reported permanent sites such as ponds and riverbeds, rather than temporary puddles and rice fields where dipping might be more difficult and detecting larvae requires more effort.

Detection and consequent reporting of late-stage *Anopheles *larvae is considered an important indicator of successful larval control in programmatic settings because it is the most practical scalable indicator for imminent emergence of adult malaria vectors. It is important to note that CORPs detection sensitivity for this key indicator was low and clearly not adequate for monitoring and management of larviciding activities. The observation that CORPs in the larviciding areas detected proportionately fewer habitats with *Anopheles *larvae, compared with those in non-larviciding areas despite the higher number in the former and even when they had reported the habitats is particularly interesting. This may be attributed to lower larval density in treated habitats and/or reduced thoroughness among individual CORPs when searching habitats as they assume sites have been treated. Moreover, biases in the perspectives and CORP supervision practices of the ward supervisors with the competing interest of being responsible for larvicide application and surveillance, may account for these trends. The fact that larval occupancy in areas with larviciding was only reduced if habitats had been found by surveillance CORPs, suggests that if surveillance CORPs did not enter a plot or detect the habitat larviciding CORPs were also less likely to enter and treat them. Although a large number of CORPs were employed and a substantive internal quality control system formed an integral part of the routine protocols of the UMCP [37], it is striking that these did not detect these substantive problems in the front-line surveillance systems. These findings call for special emphasis upon directed strategies ensuring a more compliant operational team and engagement of the community in holding these teams accountable, as well as allowing area-wide access to plots and compounds.

## Conclusion

The full true programmatic value of larviciding can only be established through evaluations of sustainable systems, which achieve much improved coverage relative to that reported here. The study has shown that unless improved access to fenced plots, and consequently detection of aquatic habitats and of larvae in them, is achieved, larviciding effectiveness will remain limited. To effectively implement larval control, we recommend that a less extensive surveillance system, focusing more on internal quality assurance based on accurate and timely reporting, be adopted. The labour-intensive and therefore expensive surveillance system implemented during the pilot phase of the UMCP [37] should be abandoned. Instead, it is recommended that rigorous external quality control of the internal process indicators used by implementers will be essential to make such monitoring systems meaningful and effective.

## Competing interests

A substantial portion of the current salary and research support for several of the investigators depends on the achievement of documented suppression of malaria transmission and infection risk by this programme through systematic larviciding. None of the funders had any role in the evaluation design, data collection, analysis, interpretation, drafting of the manuscript or decision to publish.

## Authors' contributions

PPC took the lead in designing, implementing the study, data analysis and in writing of the manuscript. NJG supported the design and implementation of the study, BS and AH participated in the implementation of various aspects of the larval surveillance and supervision of data management systems for the program. UF and GFK designed and implemented the larviciding system. UF and MT contributed substantially to drafting of the manuscript. GFK supervised all aspects of the study design, implementation, data analysis and drafting of the manuscript. All the authors have read and approved the final manuscript.

## Supplementary Material

Additional file 1 **Standardized field data collection forms**. The document presents the standardized data collection forms used for mapping and describing the habitats during the quantitative cross-sectional evaluation of the community-based larval surveillance of the UMCP, Dar es Salaam, Tanzania. Click here for file
